# ^18^ F-FDG Brain PET/MRI in Amyotrophic Lateral Sclerosis– Frontotemporal Spectrum Disorder (ALS-FTSD)

**DOI:** 10.1055/s-0043-1760762

**Published:** 2023-05-01

**Authors:** Faizullah Mashriqi, Bibhuti B. Mishra, Luca Giliberto, Ana M. Franceschi

**Affiliations:** 1 Neuroradiology Division, Department of Radiology, Northwell Health/Donald and Barbara Zucker School of Medicine, Lenox Hill Hospital, New York, United States; 2 Department of Neurology, Donald and Barbara Zucker School of Medicine at Hofstra/Northwell, The Feinstein Institutes for Medical Research. Manhasset, New York, United States

**Keywords:** amyotrophic lateral sclerosis, amyotrophic lateral sclerosis-frontotemporal spectrum disorder, ^18^ F-FDG, PET/MRI, brain PET

## Abstract

Amyotrophic lateral sclerosis (ALS) is a fatal and progressive neurodegenerative disorder involving both upper and lower motor neurons. Interestingly, 15 to 41% of patients with ALS have concomitant frontotemporal dementia (FTD). Approximately, 50% of patients with ALS can copresent with a broader set of neuropsychological pathologies that do not meet FTD diagnostic criteria. This association resulted in revised and expanded criteria establishing the ALS-frontotemporal spectrum disorder (FTSD). In this case report, we review background information, epidemiology, pathophysiology, and structural and molecular imaging features of ALS-FTSD.

## Introduction

In patients with amyotrophic lateral sclerosis (ALS), degeneration of upper and lower motor neurons ultimately leads to severe disability and eventual mortality often in the setting of respiratory failure. Notably, frontal cortex neurons are also often involved. The average survival is 3 to 5 years following diagnosis, 1 with an incidence of 1.7 per 100,000 and prevalence of 5 in 100,000. 2 Ninety percent of ALS cases are sporadic and 10% are familial, which occur secondary to any multitude of genetic mutations, including C9ORF72, SOD1, FUS, and TDP-43. 3 4 Up to 41% of patients with ALS also have frontotemporal abnormalities characterized by behavioral or cognitive dysfunction, similarly to the presented case. 4 Interestingly, up to 20% of patients with frontotemporal dysfunction can subsequently develop motor neuron disease. 4 Clinically, there is no laboratory finding that can confirm the diagnosis of ALS; therefore, it is important to exclude reversible diagnoses before considering ALS in a patient. These include multifocal neuropathy, Lyme disease, subacute combined degeneration in the setting of B12 deficiency, and thyroid disorders. 4 The pathophysiology of ALS remains unclear. While autopsy has demonstrated iron deposition in the motor cortex layer microglial cells, it remains uncertain if this is related to neuronal degeneration or ALS pathogenesis. 4

## Case Report

We present a 66-year-old male with a past medical history of hypertension, peripheral arterial disease, and polio in childhood with residual left lower extremity weakness. His family history was significant for dementia of uncertain type in both parents and his sister. At 58 years, the patient started falling due to increasing weakness in both his legs, left greater than right. His symptoms continued to progress and involved his upper extremities soon later. Anecdotally, he found it difficult to lift gallons of water or take out the trash. At the time of presentation to our clinic, the patient also endorsed slurred speech. He did not suffer from dysphagia or shortness of breath. After 2 years, the patient was diagnosed with ALS based on electromyography results and tongue fasciculation noted by ultrasound.

Three years later, the patient's symptoms continued to progress. Weakness involved the limbs, trunk, neck, and respiratory muscles, which made it difficult to ambulate safely. He was able to consume only small meals resulting in progressive weight loss. One year later, the patient began to exhibit progressive cognitive decline characterized by memory loss, behavioral changes (increased emotional lability), and increasing dysarthria. He had difficulty finding words and could not express himself over the phone. Subsequently, disorientation during the daytime and nighttime ensued. Detailed cognitive assessment was limited by the patient's fatigue and dysarthria. The assessment showed he was disoriented to time, but oriented to person and place. His short and remote memory were both impaired. He had difficulty repeating phrases and his fluency was not intact. His attention span was normal.

**Fig. 1:**
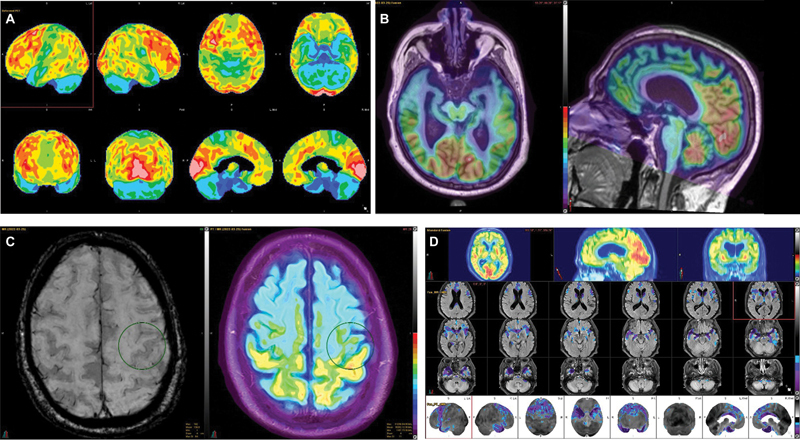
( **A** ) ^18^ F-fluorodeoxyglucose ( ^18^ F-FDG) brain positron emission tomography/magnetic resonance imaging (PET/MRI) three-dimensional stereotactic surface projection (3D-SSP) PET cortical surface metabolic maps demonstrate moderate-to-severe decreased radiotracer uptake in the frontal and anterior temporal lobes, particularly involving the superior and dorsolateral frontal regions including the supplementary motor area and frontal operculum, and also involving the anterior cingulate gyri bilaterally. ( **B** ) ^18^ F-FDG brain PET/MRI axial and coronal fused 3D T1-MPRAGE MRI-PET images demonstrate frontotemporal brain parenchymal volume loss in addition to the hypometabolism, which is particularly pronounced in the superior and dorsolateral frontal lobes, sensorimotor cortex, and anterior temporal regions and advanced for patient's age. ( **C** ) ^18^ F-FDG brain PET/MRI axial susceptibility-weighted imaging (SWI) MRI and axial fused fluid attenuated inversion recovery (FLAIR) MRI-PET images demonstrate abnormal gyriform hypointensity in the precentral gyri on SWI indicating abnormal iron deposition in the primary motor cortex, as has been described in patients with amyotrophic lateral sclerosis. ( **D** ) ^18^ F-FDG brain PET/MRI Z-score statistical maps superimposed on FLAIR axials and 3D-SSP PET demonstrate significantly decreased values in the bilateral frontal lobes including in the inferior frontal gyri, superior medial frontal gyri and supplementary motor area, temporal lobes including in the medial temporal lobes, temporal poles, amygdala, parahippocampal gyri, hippocampi and temporal operculum, bilateral caudate nuclei, middle cerebellar peduncle, pons, and medulla. Please see Table 1 for details.

^18^ F-fluorodeoxyglucose ( ^18^ F-FDG) brain positron emission tomography/magnetic resonance imaging (PET/MRI) was obtained as part of the diagnostic workup. The study demonstrated moderate-to-severe decreased radiotracer uptake in the frontal and anterior temporal lobes, particularly involving the superior and dorsolateral frontal regions including the supplementary motor area and frontal operculum, and also involving the anterior cingulate gyri bilaterally ( Fig. 1A ). There was corresponding frontotemporal brain parenchymal volume loss, particularly pronounced in the superior and dorsolateral frontal lobes, sensorimotor cortex, and anterior temporal regions, advanced for patient's age ( Fig. 1B ). There was also abnormal gyriform hypointensity in the precentral gyri on susceptibility-weighted imaging (SWI) indicating abnormal iron deposition in the primary motor cortex, as has been described in patients with ALS ( Fig. 1C ). Semiquantitative analysis using Z-scores calculated in comparison to age-matched normal controls revealed significantly decreased values in the bilateral frontal lobes including in the inferior frontal gyri, superior medial frontal gyri and supplementary motor area, temporal lobes including in the medial temporal lobes, temporal poles, amygdala, parahippocampal gyri, hippocampi and temporal operculum, bilateral caudate nuclei, middle cerebellar peduncle, pons, and medulla ( Fig. 1D and Table 1 ).

**Table 1:** Semiquantitative Z-scores in tabular format

Atlas	Structure	Z-Score	L Z-Score	R Z-Score	L-R% Diff	L-R% Diff Z-Score
Single brain atlas	Temporal pole	**−9.13**	**−6.36**	**−8.56**	26.15	3.79
Single brain atlas	Amygdala	**−7.39**	**−5.28**	**−8.43**	28.26	8
Single brain atlas	Caudate	**−7.22**	**−6.93**	**−7.12**	−3.87	−1.56
MM probabilistic atlas	Amygdala	**−7**	**−5.47**	**−7.08**	25.4	4.38
MM probabilistic atlas	Caudate	**−6.8**	**−6.96**	**−6.25**	−8.55	−3.04
Single brain atlas	Anterior cingulate gyrus	**−5.03**	**−4.37**	**−5.83**	12.43	4.09
Single brain atlas	Cingulate gyrus	**−4.71**	**−4.4**	**−4.41**	−0.27	0.51
Single brain atlas	Superior medial frontal gyrus	**−3.58**	**−2.58**	**−4.3**	10.34	3.69
Single brain atlas	Parahippocampal gyrus	**−3.44**	**−2.17**	**−3.93**	13.41	2.91
Single brain atlas	Medial temporal lobe	**−3.35**	**−2.02**	**−3.92**	15.54	4.32
Single brain atlas	Supplementary motor area	**−3.34**	**−2.62**	**−3.47**	4.03	0.19
Single brain atlas	Middle frontal gyrus	**−3.28**	**−2.6**	**−3.45**	2.15	0.99
MM probabilistic atlas	Medial temporal lobe	**−3.19**	**−2.04**	**−3.51**	14.08	3.57
Single brain atlas	Superior frontal gyrus	**−3.18**	**−3.2**	**−2.7**	−2.89	−0.28
Single brain atlas	Inferior temporal gyrus	**−3.09**	**−1.78**	**−3.39**	6.83	1.24
Single brain atlas	Hippocampus	**−3.03**	**−1.39**	**−3.67**	19.25	4.28
Single brain atlas	Inferior frontal gyrus, pars opercularis	**−3.03**	**−4.06**	−1.07	−14.55	−3.98
Single brain atlas	Subcallosal area	**−3**	**−3.33**	**−2.64**	−9.8	−2.87
Single brain atlas	Frontal lobe	**−2.93**	**−2.61**	**−2.66**	0.44	0.03
MM probabilistic atlas	Hippocampus	**−2.93**	**−1.75**	**−3.23**	15.36	3.09
MM probabilistic atlas	Parahippocampal gyrus	**−2.73**	**−1.66**	**−2.84**	11.42	1.59
Single brain atlas	Temporal operculum	**−2.69**	**−1.57**	**−3.23**	7.08	1.62
MM probabilistic atlas	Basis pontis	−2.49	N/A	N/A	N/A	N/A
Single brain atlas	Posterior cingulate gyrus	**−2.41**	**−2.42**	**−2.03**	4.42	0.36
Single brain atlas	Medulla	−2.16	N/A	N/A	N/A	N/A
Single brain atlas	Inferior frontal gyrus	**−2.15**	**−2.65**	−1.36	−3.01	−1.71
Single brain atlas	Basis pontis	−2.13	N/A	N/A	N/A	N/A
Single brain atlas	Temporal lobe	**−1.94**	−1.14	**−1.9**	−0.04	0.57
MM probabilistic atlas	Posterior cingulate gyrus	**−1.77**	−2.02	−1.64	4.01	0.4
Single brain atlas	Inferior cerebellar peduncle	**−1.73**	−0.96	**−2.16**	36.31	2.58
Single brain atlas	Middle cerebellar peduncle	−1.61	−1.75	−1.36	−2.25	−0.69
Single brain atlas	Inferior frontal gyrus, pars orbitalis	−0.9	−2.41	−0.01	2.5	−1.91

Abbreviation: N/A, not available. Bold values indicate statistically significant negative values compared with age-matched normal controls.

These findings are compatible with amyotrophic lateral sclerosis-frontotemporal spectrum disorder (ALS-FTSD), with the latter component becoming more evident clinically in recent months. Importantly, in spite of the limitations of the cognitive exam, due to advanced motor features, the presence of emotional lability and erratic behavior was well correlated with the finding of atrophy and hypometabolism in the frontal and anterior temporal regions. 5 Genetic testing was performed, and resulted negative for a large panel of mutations, including c9Orf72 (ALS2, ANG, CHCD10, FUS, GRN, HNRNPA2B1, MAPT, OPTN, PFN1, PRPH, SETX, SLC52A3, SOD1, SPG11, SQSTM1, TAF15, TARDBP, TBK1, TUBA4A, UBQLN2, VAPB, VCP, MATR3). No further collateral information is available regarding the reported family history of dementia. The above leaves open the possibility of a sporadic case of ALS-FTSD.

## Discussion

Imaging plays an important role in ruling out structural pathology that may otherwise mimic ALS by impinging on the motor tracts. MRI is the preferred imagining modality for ruling out such structural pathology. As previously discussed, there is iron deposition in the primary motor cortex. Susceptibility-weighted imaging (SWI) takes advantage of the paramagnetic properties of iron and other hemorrhagic products that result in blooming artifact. In ALS, this low signal on SWI is apparent in the primary motor cortex ( Fig. 1C ). Recent advances have enabled quantification of this deposition with susceptibility mapping in parts per million. 4 6 Advanced cases of iron deposition in the primary motor cortex may even result in low signal on conventional T2-weighted images. As expected, bulbar onset ALS may show increased iron deposition in the facial region of the primary motor cortex. 4 Increased T2-fluid attenuated inversion recovery signal in the corticospinal tract is specific to ALS, but not as sensitive as iron deposition in the precentral gyri. 4 7 MR spectroscopy demonstrates decreased N-acetyl-aspartate metabolite in the primary motor cortex. 4 8 9 Diffusion tensor imaging has demonstrated that the most apparent changes in ALS patients occur in the corticospinal tract. Additional areas of involvement include the frontal white matter, brain stem, and hippocampal regions. 4 10 Morphometry in ALS demonstrates primary motor cortex atrophy. Frontal, temporal, and parietal atrophy has also been described.

FDG-PET in ALS was first studied in the late 1980s and demonstrated decreased radiotracer uptake in the primary motor cortex and prefrontal cortex. 11 Interestingly, ALS-associated PET findings that suggest involvement beyond the precentral gyrus and primary motor cortex predate the acceptance of ALS as part of the ALS-FTSD continuum. 11 12 More recent studies in the literature have reported decreased ^18^ F-FDG uptake in the premotor cortex and frontal cortex, and increased uptake in the brain stem. 4 11 Increased brain stem radiotracer uptake is hypothesized to be related to subcortical gliosis. 4 Furthermore, data suggests that decreased radiotracer uptake in the prefrontal and anterior temporal cortices is associated with shorter survival. 4 The spinal cord has also recently been evaluated by ^18^ F-FDG PET in ALS patients, demonstrating increased uptake in the cervical spinal cord. 11 In fact, spinal cord hypermetabolism was inversely correlated with patient's overall survival, enabling assessment of disease severity. 13 This is in contrast to ALS mimics, which typically demonstrate relatively lower spinal cord radiotracer uptake. 4 In a 2020 study of 98 patients with suspected ALS, Van Weehaeghe et al demonstrated that brain metabolism was similar in ALS and variant motor neuron diseases including primary lateral sclerosis and progressive muscular atrophy. On the other hand, ALS patients demonstrated higher cervical and thoracic spine metabolism compared with ALS mimics with and without progressive muscular atrophy or primary lateral sclerosis. 14 Spinal and muscular bulbar muscular atrophy, also known as Kennedy's disease, is another major differential diagnosis for motor neuron disorders. While studies have demonstrated frontal lobe hypometabolism in Kennedy's disease, the role of ^18^ F-FDG PET remains unclear in differentiating it from ALS. 15 Hirayama disease, a type of muscular atrophy, may also mimic ALS. There is limited data about the use of ^18^ F-FDG PET in differentiating ALS from Hirayama disease, although a recent report has demonstrated similar cervical spine hypermetabolism. 16 Recently, the literature has shown that PET imaging may play a role in determining ALS prognosis. Studies have demonstrated an association between mortality rate and frontotemporal hypometabolism as well as spinal cord hypermetabolism on ^18^ F-FDG PET. 11 13 The role of ^18^ F-FDG PET in determining the likelihood of cognitive impairment and disease progression remains unclear and warrants future studies. 11

Additional PET radiotracers have been studied in ALS patients, taking advantage of GABAergic, dopaminergic and serotonergic functions, as well as oxidative stress. The GABA _A_ receptor ligand ^11^ C-Flumazenil has been used to demonstrate cortical hyperexcitability in ALS patients. Decreased radiotracer uptake has been demonstrated in the frontal lobes and anterior cingulate gyrus. 4 11 The serotonin ligand ^11^ C-WAY100635 binds to 5-HT1a receptors in layers III and V in the cortex. Studies have demonstrated decreased radiotracer uptake in the precentral gyrus (primary motor cortex), cingulate, parahippocampal, and fusiform gyri in ALS patients. 11 Postmortem analysis has demonstrated substantia nigra degeneration in ALS patients raising the possibility of overlap with Parkinson's disease. Data regarding this dopaminergic dysfunction in ALS patients is contradictory. Various radiotracers ( ^18^ F-fluorodopa and ^11^ C-N-methylspiperone) show no significant difference between ALS and control patients. Other radiotracers ( ^18^ F-Fallypride) demonstrated decreased dopaminergic cortical activity in ALS patients without a history of parkinsonism. One mechanism proposes involvement of cortical dopaminergic neurons (rather than striatal) in ALS. 11 PET utilizing ^62^ Cu-ATSM, a ligand similar to superoxide dismutase, targets brain regions that are hypoxic or undergoing oxidative stress. As expected, increased tracer accumulation has been demonstrated in the motor cortices, paracentral lobules, and in the right superior parietal lobule. 11

Unfortunately, current treatment paradigms do not offer significant clinical benefit. U.S. Food and Drug Administration-approved drugs for ALS include riluzole and edaravone. The mechanism of action of edaravone involves oxidative stress reduction and that of riluzole involves decreasing excessive neuronal firing. 1 Supportive therapy includes minimizing aspiration with cough assist devices and ventilatory support. Future directions, including gene therapy, are under investigation. Such therapy includes delivery of therapeutic genes or silencing elements via viruses. 1

It is important to note that 15 to 41% of patients with ALS can also have frontotemporal dementia (FTD). 4 However, approximately 50% of patients with ALS can present with a broader set of heterogeneous neuropsychological pathologies that do not necessarily meet FTD diagnostic criteria. This association resulted in revised and expanded criteria establishing the ALS-FTSD spectrum. 12 The ALS-FTSD spectrum includes ALS-behavioral impairment, ALS-cognitive impairment, ALS-FTD, and ALS-dementia. Interestingly, the broadened criteria—as proposed in a consensus conference in the summer of 2015—emphasize an overlap between neurologic deficits but do not necessarily infer a continuum between these subtypes. 12 A diagnosis of ALS-cognitive impairment is established when there is evidence of executive or language dysfunction. ALS-behavioral impairment is characterized by the presence of apathy and the presence of behavioral symptoms (including loss of sympathy/empathy, psychotic symptoms, and hyperorality). ALS-combined cognitive and behavioral impairment is diagnosed when criteria for the previous two diagnoses are both met. ALS-FTD is characterized by the presence of cognitive dysfunction that is compatible with a diagnosis of FTD. 12 Noteworthy, whether or not the ALS-FTSD subtypes that do not meet FTD criteria ultimately progress to ALS-FTD remain uncertain. 12

Despite extensive strides in our basic science knowledge of ALS and ALS-FTSD, this complex spectrum of pathology remains difficult to diagnose and manage. Future advances in diagnostic imaging modalities, genomics, and clinical pharmaceuticals may further shed light on the pathophysiology and management of this neurodegenerative disease spectrum.

## References

[1] Brown R H Al-Chalabi A Amyotrophic lateral sclerosis N Engl J Med 2017 377 02 162 172 28700839 10.1056/NEJMra1603471

[2] Oskarsson B Gendron T F Staff N P Amyotrophic lateral sclerosis: an update for 2018 Mayo Clin Proc 2018 93 11 1617 1628 30401437 10.1016/j.mayocp.2018.04.007

[3] Mejzini R Flynn L L Pitout I L Fletcher S Wilton S D Akkari P A Als genetics, mechanisms, and therapeutics: where are we now? Front Neurosci 2019 13 1310 10.3389/fnins.2019.01310 31866818 PMC6909825

[4] Schweitzer A Tsiouris A J Amiotrophic lateral sclerosis Springer Nature Switzerland 2022. ISBN 978–3-030–82366–5. eISBN 978–3-030–82367–2. 78–3-030–82367–2. 10.1007/978-3-030-82367-2

[5] Kumfor F Irish M Hodges J R Piguet O Frontal and temporal lobe contributions to emotional enhancement of memory in behavioral-variant frontotemporal dementia and Alzheimer's disease Front Behav Neurosci 2014 8 225 10.3389/fnbeh.2014.00225 25009480 PMC4067999

[6] Wang Y Spincemaille P Liu Z Clinical quantitative susceptibility mapping (QSM): biometal imaging and its emerging roles in patient care J Magn Reson Imaging 2017 46 04 951 971 28295954 10.1002/jmri.25693 PMC5592126

[7] Vázquez-Costa J F Mazón M Carreres-Polo J Brain signal intensity changes as biomarkers in amyotrophic lateral sclerosis Acta Neurol Scand 2018 137 02 262 271 29082510 10.1111/ane.12863

[8] Govind V Sharma K R Maudsley A A Arheart K L Saigal G Sheriff S Comprehensive evaluation of corticospinal tract metabolites in amyotrophic lateral sclerosis using whole-brain 1H MR spectroscopy PLoS One 2012 7 04 e35607 10.1371/journal.pone.0035607 22539984 PMC3335096

[9] first Neuroimaging Symposium in ALS (NISALS) Turner M R Grosskreutz J Kassubek J Towards a neuroimaging biomarker for amyotrophic lateral sclerosis Lancet Neurol 2011 10 05 400 403 21511189 10.1016/S1474-4422(11)70049-7

[10] Neuroimaging Society in ALS (NiSALS) DTI Study Group Müller H P Turner M R Grosskreutz J A large-scale multicentre cerebral diffusion tensor imaging study in amyotrophic lateral sclerosis J Neurol Neurosurg Psychiatry 2016 87 06 570 579 26746186 10.1136/jnnp-2015-311952

[11] Chew S Atassi N Positron emission tomography molecular imaging biomarkers for amyotrophic lateral sclerosis Front Neurol 2019 10 135 10.3389/fneur.2019.00135 30881332 PMC6405430

[12] Strong M J Abrahams S Goldstein L H Amyotrophic lateral sclerosis - frontotemporal spectrum disorder (ALS-FTSD): revised diagnostic criteria Amyotroph Lateral Scler Frontotemporal Degener 2017 18(3-4):153 174 28054827 10.1080/21678421.2016.1267768 PMC7409990

[13] Marini C Morbelli S Cistaro A Interplay between spinal cord and cerebral cortex metabolism in amyotrophic lateral sclerosis Brain 2018 141 08 2272 2279 30730551 10.1093/brain/awy152 PMC6061793

[14] Van Weehaeghe D Devrome M Schramm G Combined brain and spinal FDG PET allows differentiation between ALS and ALS mimics Eur J Nucl Med Mol Imaging 2020 47 11 2681 2690 32314027 10.1007/s00259-020-04786-y

[15] Lai T H Liu R S Yang B H Cerebral involvement in spinal and bulbar muscular atrophy (Kennedy's disease): a pilot study of PET J Neurol Sci 2013 335(1-2):139 144 24120273 10.1016/j.jns.2013.09.016

[16] Cabona C Benedetti L Delucchi S Cervical alterations in Hirayama disease: an MRI and FDG-PET combined approach Clin Transl Imaging 2021 9 117 119

